# Red Light Enhances Biomass and Bioactive Compounds Through Photosynthetic Acclimation in *Anabaena variabilis*

**DOI:** 10.3390/md24060221

**Published:** 2026-06-19

**Authors:** Carol Ostojic, María Robles, Lidia Martín-Gordillo, David Fernández, Riccardo Gava, Carlos Vílchez

**Affiliations:** 1 Biotechnology of Extremophiles Lab, CIDERTA, CIQSO-Centro de Investigación en Química Sostenible, University of Huelva, 21071 Huelva, Spain; carolostojic@bioplagen.com (C.O.); lidia.martin@dqcm.uhu.es (L.M.-G.); frdavid14@gmail.com (D.F.); 2 New Biosecurity Technologies S.L., Av. Castilleja de la Cuesta, 20-22, Bollullos de la Mitación, 41110 Seville, Spain; riccardogava@bioplagen.com

**Keywords:** cyanobacteria, PAR-red light, biomass productivity, photosynthesis, phycobiliproteins

## Abstract

Light irradiance and spectral quality are key environmental factors that influence the growth, photosynthetic performance, and metabolic responses of cyanobacteria. In this study, the effects of increasing white and PAR-red light irradiances on *Anabaena variabilis* were evaluated in repeated-batch cultures, focusing on photosynthetic efficiency, biomass productivity, and the modulation of antioxidant systems, while cultures maintained under constant irradiance were used as control. Results showed that *A. variabilis* can maintain photosynthetic efficiency, as indicated by F_V_/F_M_ values, within the optimal range for healthy cultures despite variations in light conditions. PAR-red light, in particular, enhanced biomass productivity and induced stronger photoacclimation responses compared to white light. Moreover, analysis of chlorophyll fluorescence (JIP parameters) revealed that photosynthetic machinery adapts to increased irradiance by modulating energy fluxes. Dissipated energy (DI_0_/RC) increases by 4.5-fold under increasing PAR-red light with respect to control cultures, which suggests that PAR-red light promotes thermal dissipation of excess absorbed energy at the phycobilisome level, independently of and complementarily to, the increase in light-harvesting antenna pigments (chlorophylls and phycobiliproteins), thereby reducing the net oxidative pressure in the electron transport chain. The increase in photosynthetic pigments reflects an adaptive adjustment to optimize light harvesting under red light, with a phycocyanin content of 123 mg·g^−1^ biomass, 30% higher than that obtained in control culture. Overall, *A. variabilis* demonstrated a robust capacity to acclimate increasing light irradiance and varying light quality through coordinated photoacclimation and antioxidant responses, in repeated-batch cultures. These findings highlight its physiological flexibility, which can be properly driven to maximize the production of valuable bioactive compounds, particularly phycobiliproteins such as phycocyanin, with applications in biotechnology.

## 1. Introduction

Light is one of the determining environmental factors for photosynthetic microorganisms, as it not only provides the energy necessary for photosynthesis but also acts as a signal that regulates key cellular processes [1]. In the specific case of cyanobacteria, both irradiance and the spectral range of light directly modulate photosynthetic efficiency, biomass accumulation, and the production of bioactive compounds with industrial applications [2]. However, excessive exposure to inappropriate wavelengths can induce photoinhibition, damage to the photosystem, and overproduction of reactive oxygen species (ROS), generating a redox imbalance harmful to the cell [3]. That is, under these conditions, the photosynthetic electron transport chain (ETC) becomes saturated and maximum levels of NADPH are generated, which results in increased NAD(P)H/NAD(P)^+^ ratios [4]. At this point, this excess energy reacts with O_2_, favoring the production of the first ROS species, singlet oxygen (^1^O_2_) [5]. This species gives rise to more damaging ROS species such as the superoxide anion (O_2_^−^), hydrogen peroxide (H_2_O_2_) and the hydroxyl radical (·OH) [6,7]. These species play a relevant role as signaling molecules which trigger the biosynthesis of antioxidant molecules and the expression of antioxidant enzymes [8]. If ROS are present in excess, they can cause severe oxidative damage by reacting with lipids, proteins and nucleic acids, among other biomolecules, leading to irreversible alterations of cellular structures [9]. Furthermore, they cause damage to the oxidative centers of the oxygen-evolving complex of water photolysis and deactivate the ETC, resulting in a loss of photosynthetic activity and cell death [10,11].

In this context, it is essential to understand how the photosynthetic machinery of cyanobacteria is affected by ROS-formation inducing factors, including excess light, and therefore how to manage cultures accordingly in order to minimize productivity losses. The photosynthetic machinery of cyanobacteria is mainly composed of photosystems I and II (PSI and PSII), together with the cytochrome b_6_f complex, ATP synthase, and phycobilisomes, structures that allow the efficient capture of photons and transfer of light energy to the reaction centers [12]. The functional efficiency of these complexes can be evaluated through chlorophyll *a* fluorescence, particularly by analyzing the polyphasic fluorescence induction curve (OJIP), also known as the JIP test, which reflects the sequential reduction events in the photosynthetic electron transport chain. This approach has been applied in studies with cyanobacteria to characterize parameters such as the maximum quantum yield (F_V_/F_M_), the energy absorbed by the active reaction center (ABS/RC), the electron transport rate per reaction center (ET_0_/RC), the energy dissipated per reaction center (DI_0_/RC), and the basal photosynthetic performance index (PI_ABS_), among others, which allow the evaluation of PSII quantum efficiency and energy balance under stress conditions [13]. These parameters allow exploring the adjustment mechanisms that cyanobacteria have developed in response to changes in the light environment.

Cyanobacteria have developed photoacclimation mechanisms that allow them to adapt to changes in light intensity and spectral range. One of the most studied is far-red light photoacclimation mechanism (FaRLiP), in which alternative chlorophylls such as chlorophyll *f* and *d* are synthesized to broaden the absorption spectra towards wavelengths > 700 nm [14]. This mechanism includes the structural remodeling of photosystems and the expression of alternative genes, allowing efficient photosynthesis in environments with filtered or low-energy light, such as microbial mats or endolithic rocks [15]. Several studies have shown that the spectral range of light, particularly red light within the PAR spectrum (600–700 nm), is more efficient than white or blue light in stimulating photosynthesis, due to its coincidence with the absorption peaks of chlorophyll *a* and phycobiliproteins [16]. These observations help explain why certain qualities of light, such as red, are particularly effective for these microorganisms.

In species such as *Phormidium* sp., *Cyanothece* sp., *Nostoc* sp., and *Microcystis aeruginosa*, red light has been shown to stimulate greater biomass and pigment production, while white light induces mixed responses depending on the intensity [17]. On the other hand, the accumulation of antioxidant compounds such as phycobiliproteins and phenolic compounds, and other biotechnologically relevant molecules such as lipids, has been associated with defense mechanisms against high irradiance or energy imbalance in the electron transport chain [18]. These metabolites, in addition to fulfilling essential functions in cellular photoprotection, have high added value for their application in industrial sectors such as the agricultural biotechnology, nutraceutical, cosmetics, and pharmaceutical industries, where they are used as natural colorants and antioxidant compounds in various formulations [19]. Given this background, studying specific species that meet those physiological and biotechnological qualities becomes of scientific and applied relevance.

Although *Anabaena* is predominantly a freshwater genus, *A. variabilis* ATCC 29413 tolerates NaCl concentrations up to 100 mM [20], making it suitable for mild-salinity biotechnological applications. Beyond its physiological robustness, *A. variabilis* produces a range of metabolites of direct relevance to marine drug potential and/or biomedical applications, including neurotoxins with anesthetic activity [21], phycobiliproteins with documented antioxidant, anti-inflammatory, anticancer, neuroprotective and antiviral activities [22], as well as lipids and phenolic compounds with emerging pharmaceutical interest [19,23]. The biotechnological relevance of *Anabaena* extends beyond phycobiliproteins to encompass compounds traditionally classified as marine-derived drugs with documented pharmaceutical relevance. Saxitoxins and their analogues, including neosaxitoxin, currently under clinical evaluation as a long-acting local anesthetic [21], were originally characterized as paralytic shellfish toxins from marine dinoflagellates (*Alexandrium* spp., *Gymnodinium catenatum*) and can be considered classic examples in the marine natural products pharmacopoeia. *Anabaena circinalis* and *Anabaena lemmermannii* biosynthesize the same molecular scaffolds [21], positioning *Anabaena* as a controllable, scalable production bioplatform that can compete with (and potentially replace) marine dinoflagellate sources, which can be difficult to cultivate at industrial scale and yield variable toxin profiles. Similarly, phycobiliproteins produced by *A. variabilis* occupy the same therapeutic space as those extracted from the marine red microalga *Porphyridium cruentum*, a major commercial source of pharmaceutical-grade phycoerythrin. Anabaenopeptins (cyclic hexapeptides produced via non-ribosomal peptide synthetase pathways, first isolated from *Anabaena* sp.) are potent inhibitors of carboxypeptidases A and B and of serine/threonine phosphatases PP1 and PP2A, making them potential candidate scaffolds for the development of inhibitors relevant to metabolic and oncological disorders [23]. These examples underscore the direct relevance of *Anabaena* spp. as a platform for production of marine drugs of pharmaceutical interest.

*A. variabilis* is a differentiated filamentous cyanobacterium capable of fixing atmospheric nitrogen through specialized heterocysts, even under varying nitrogen conditions [24]. Its physiological robustness to variable environmental conditions, along with its ability to produce pigments with antioxidant activity, make it an ideal model microorganism for studying the influence of light on the regulation of photosynthetic and biosynthetic processes [25]. Although studies have explored its response to ultraviolet radiation and different carbon sources [26], information on how *A. variabilis* responds to variations in irradiance combined with different light spectral ranges remains limited, particularly regarding the activation of antioxidant mechanisms and photoacclimation. Therefore, a detailed evaluation of these responses in *A. variabilis* may provide valuable information for future applications including scaling up processes.

Understanding the effects of the spectral range and intensity on the physiology of *A. variabilis* may be essential for optimizing its use in low-energy cultivation systems and for potential applications in agriculture, water treatment, or antioxidant formulations. Furthermore, the integrated analysis of photosynthetic efficiency using JIP parameters, together with the quantification of bioactive compounds, allows for the identification of optimal lighting conditions that maximize productivity without inducing, or minimizing, oxidative damage. Prior studies on *A. variabilis* have primarily addressed several light-related issues including, among others, pigment dynamics under outdoor solar irradiance cycles, responses to chromatic adaptation, photosynthetic response under red vs. fluorescent light, and photoinhibition under high-intensity white light [27]. However, to our knowledge, no prior study has evaluated the comparative effect of monochromatic PAR-red versus white light at increasing irradiances on the full set of photosynthetic energy fluxes (JIP test parameters) together with the antioxidant biomolecule profile of *A. variabilis* in a repeated-batch cultivation regime; this study can help approach conditions for scaling up the production of valuable molecules in the cyanobacterium. Accordingly, the objective of this work is to evaluate the effect of light irradiance and spectral range (white vs. red) on growth, photosynthetic efficiency, and the accumulation of bioactive compounds in *A. variabilis*, with an emphasis on the mechanisms of photoacclimation and response to excessive light irradiance.

## 2. Results

This paper aimed to evaluate the effect that different light irradiances and qualities have on the growth and production of valuable molecules in the cyanobacterium *A. variabilis*. Cultivation of *A. variabilis* under either white or PAR-red light was performed in repeated-batch mode, maintaining the cultures in optical densities (OD_750_) ranging from 1.5 to 3. In addition, light irradiance was increased after each repeated-batch cycle. After culture dilution, culture turbidity is lower; therefore, the light that reaches the cells is even higher. Control cultures were established for each light quality without increasing light irradiance.

Specific irradiance ranges were selected, from 120 to 250 μmol photon·m^−2^·s^−1^ for white light and 250 to 450 μmol photon·m^−2^·s^−1^ for PAR-red light. Light irradiances were selected to span a sub-inhibitory range for each light quality, informed by preliminary observations on culture viability and consistent with the photoacclimation capacity of cyanobacteria under moderate to moderately high irradiances [27]. The different ranges applied for white and PAR-red light reflect the distinct tolerance of *A. variabilis* to each spectral quality, as confirmed by the F_V_/F_M_ values maintained throughout this study (Figure S1). This paper analyzes the adaptability of the cyanobacterium to increasing light irradiances under different light qualities (white and red), together with the antioxidant mechanisms that cope with excess light.

### 2.1. Effect of Light Irradiance and Quality on Growth and Photosynthetic Viability of A. variabilis

This section focuses on the growth and photosynthetic viability of the cyanobacterium *A. variabilis* exposed to light of different irradiances and qualities. As explained above, the cyanobacterium was grown under repeated-batch mode, in which, after each cycle, light irradiance was increased, while a culture without increasing light irradiance was selected as the control culture for each light quality. The growth dynamics of the cyanobacterium are shown in the following figure using the parameters optical density and biomass productivity.

**Figure 1:**
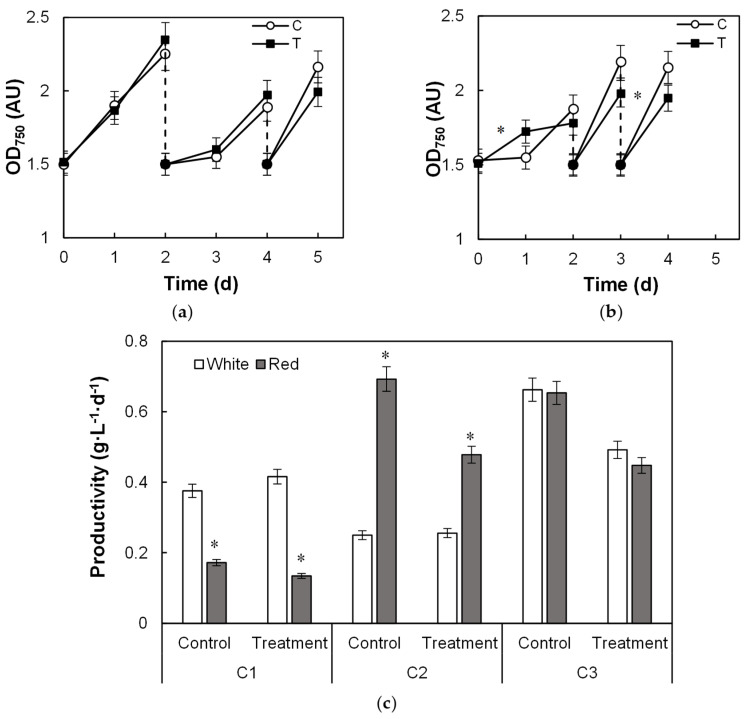
Time-dependent evolution of growth of repeated-batch cultures of *A. variabilis* as a function of light intensity and quality: (**a**) white light and (**b**) red light; and (**c**) biomass productivity. Discontinuous line represents each cycle of repeated-batch cultivation, represented in (**c**) as cycle 1 (C1), cycle 2 (C2) and cycle 3 (C3). Legend symbols: Control culture (C), (-○-) and cultivation under increased light irradiance, treatment (T), (-■-). OD_750_ values are expressed in absorption units (AU), dimensionless absorbance units proportional to culture turbidity. More details can be found in Section 4. (*) Represents the significant differences of both increasing light irradiance treatments with respect to control irradiance (**a**,**b**) and PAR-red light with respect to white light irrespective of light irradiance (**c**) with 95% confidence.

Figure 1 analyzes the growth of *A. variabilis*, measured as optical density, as a function of light irradiance and quality. As explained above, light irradiance was increased after each repeated-batch cycle, with the exception of control culture. Therefore, after each dilution, the light experienced by each cell was even higher. The discontinuous line represents each cycle of repeated-batch cultivation. It is important to note that the three consecutive repeated-batch cycles (C1, C2, C3) do not represent biological replicates of identical conditions, but rather sequential physiological states of the culture under progressive adaptation to the imposed conditions (treatment). Minor inter-cycle fluctuations in control cultures reflect the dilution-recovery dynamics inherent to repeated-batch cultivation, and were confirmed statistically non-significant (*p* > 0.05). According to the growth curves presented in Figure 1a,b, increasing light irradiance after each repeated-batch cycle does not generate significant changes in optical density values, with the exception of PAR-red light cultivation (cycles C2 and C3), in which lower optical density values were obtained under increased light irradiance.

For a better understanding of the differences observed in the growth of *A. variabilis*, Figure 1c analyzes the biomass productivity of the cyanobacterium. From the analysis, it can be inferred that light quality can be modulated to enhance the productivity of *A. variabilis*. That is, irrespective of the light quality to which the cyanobacterium was exposed, similar final biomass productivities of about 0.6 g·L·d^−1^ were obtained. However, PAR-red light seems to be more advantageous for *A. variabilis* as biomass productivity peaked earlier, in cycle 2 (C2), than in the cultures exposed to white light. This fact would reduce both the economic and energetic production costs of the cyanobacterium, resulting in a beneficial outcome for large-scale production. Lower light irradiance seems to enhance the growth of the cyanobacterium as higher biomass productivities were generally obtained in the control culture, contributing, eventually, to reducing production costs.

The following figures focus on understanding photosynthetic mechanisms developed by the cyanobacterium to cope with increasing white or red-light irradiances.

**Figure 2:**
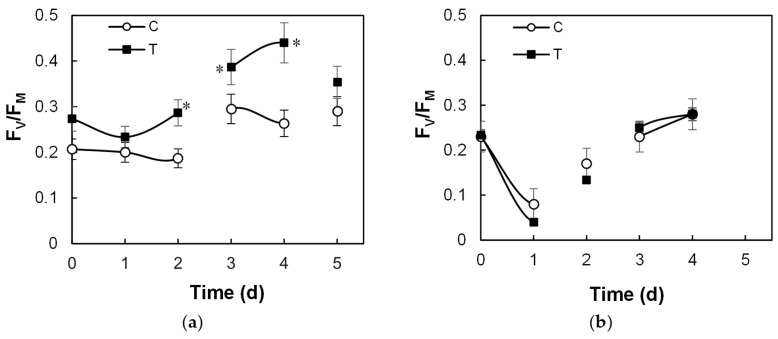
Time-dependent evolution of photosynthetic efficiency, measured as quantum yield (F_V_/F_M_), of repeated-batch cultures of *A. variabilis* as a function of light intensity and quality: (**a**) white light and (**b**) red light. Discontinuous line represents each cycle of repeated-batch cultivation. Legend symbols: Control culture (C), (-○-) and cultivation under increased light irradiance, treatment (T), (-■-). More details can be found in Section 4. (*) Represents the significant differences of increasing light irradiance treatments with respect to control irradiance with 95% confidence.

Figure 2 analyzes the photosynthetic viability of *A. variabilis*, measured as the photosynthetic efficiency of PSII (F_V_/F_M_), quantum yield (Qy), as a function of light irradiance and quality. According to the figure, exposure of the cyanobacterium to increasing white light irradiance resulted in higher Qy values with respect to the control (white light) culture, whereas a similar pattern (below 0.3), irrespective of the light irradiance to which *A. variabilis* was exposed, was obtained under PAR-red light. A decrease in Qy values was observed during the first cycle of repeated-batch cultivation under PAR-red light, and after that, a recovery in Qy values was observed.

**Figure 3:**
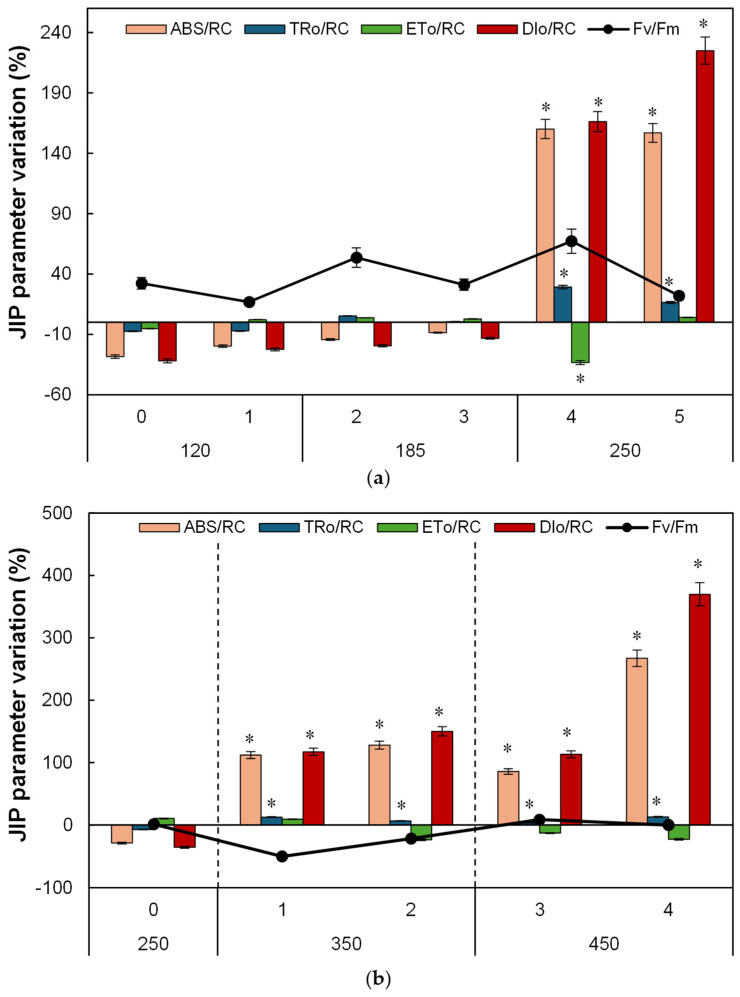
Time-dependent evolution of selected JIP parameters of *A. variabilis* cultures grown under repeated bath cultivation and subjected to different light irradiances and quality: (**a**) white light and (**b**) PAR-red light. JIP parameter data were normalized to those obtained for the control culture for each light quality. X-axis legend: time (days, top legend) and light irradiance (µmol of photon·m^−2^·s^−1^, bottom legend). Legend symbols: ABS/RC: energy absorbed by reaction center, TR_0_/RC: energy trapped by reaction center, ET_0_/RC: electronic transport by reaction center, DI_0_/RC: energy dissipated by reaction center, F_V_/F_M_: photosynthetic efficiency of photosystem II. (*) Represents the significant differences of all treatments with respect to day 0 with 95% confidence.

The following figures further examine how irradiance affects the photosynthetic machinery of the cyanobacterium. Briefly, photons are absorbed by the pigments of the antennas (ABS, absorption flux) resulting in their excitation. A part of the excitation energy is transferred to the reaction center (RC), referred to as the trapped flux (TR), while the other part is dissipated in the form of fluorescence or heat (DI, dissipated energy). The trapped flux (TR) is converted in the RC into redox energy, leading to electron transport (ET) and, consequently, photochemistry, which ultimately leads to CO_2_ fixation [28]. Figure 3 shows the time-dependent evolution of these JIP parameters in *A. variabilis* cultures grown under repeated-batch cultivation as a function of light irradiance and quality. Each cycle of repeated-batch cultivation is represented in the figure by dashed vertical lines. The data shown in the figure were normalized to the control culture data, calculated as follows: 100 × (treatment_data − control_data)/control_data.

As observed in Figure 2, an increase in the photosynthetic efficiency of photosystem II (F_V_/F_M_) with respect to the control culture was obtained in cultures grown under white light, irrespective of the repeated-batch cycle and light irradiance to which the culture was exposed (Figure 3a). Regarding specific energy flux (ABS/RC, TR_0_/RC, ET_0_/RC and DI_0_/RC), variations of no more than 25% rarely occurred with respect to the control culture during the first and second cycle of repeated-batch cultivation corresponding to light irradiances of 120 and 185 µmol of photon·m^−2^·s^−1^. However, during the third cycle of repeated-batch cultivation, a significant increase in ABS/RC and DI_0_/RC was obtained coinciding with the increase in light irradiance to 250 µmol of photon·m^−2^·s^−1^, while, interestingly, slight variations were obtained in TR_0_/RC and ET_0_/RC with respect to the control culture.

Contrary to the results obtained under white light irradiances, Figure 3b shows a similar photosynthetic efficiency of photosystem II over time in *A. variabilis* cultures grown under PAR-red light with respect to the control culture, irrespective of the light irradiance. Additionally, a progressive time-dependent increase was obtained in ABS/RC and DI_0_/RC as light irradiance increased, reaching the maximum values at the highest irradiance tested, of 450 µmol of photon·m^−2^·s^−1^. Like Figure 3a, slight variations were obtained in TR_0_/RC and ET_0_/RC with respect to the control culture.

**Figure 4:**
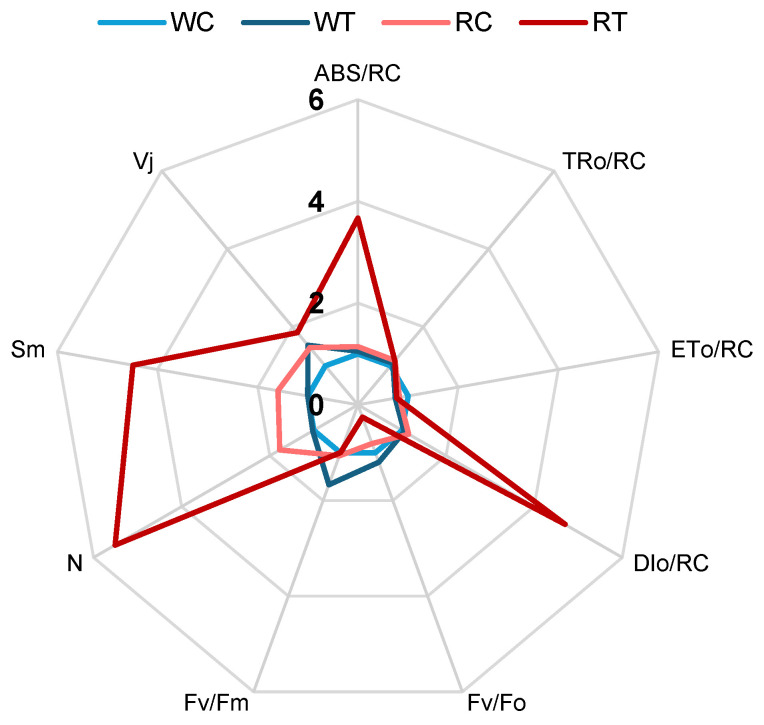
Comparison of selected JIP parameters obtained for *A. variabilis* during the last cycle of repeated batch cultivation (cycle 3, C3) as a function of light irradiance and quality. Legend symbols: white light control (WC, light blue), white treatment (WT, dark blue), PAR-red control (RC, light red) and PAR-red treatment (RT, dark red).

The results obtained in *A. variabilis* cultures grown under increasing light irradiances, irrespective of light quality, as discussed in detail in Section 3, should be interpreted as a possible activation of photoacclimation mechanisms to dissipate part of the absorbed energy. Nevertheless, considering that the greatest variations in the JIP parameters occurred during the third cycle of repeated-batch cultivation, Figure 4 focuses on a detailed analysis and comparison of the variation of selected JIP parameters related to primary photochemistry and specific energy fluxes during day 4 (third cycle of repeated-batch). The values obtained were normalized with respect to those obtained in the control culture grown under white light; thus, in Figure 4, the regular shape of the radial plot indicates the data of the aforementioned control culture. According to Figure 4, after increasing PAR-red light irradiances in *A. variabilis*, the spider plot shape greatly differs from that of the control culture. Therefore, the photosynthetic machinery of the cyanobacterium was more affected under increased PAR-red light irradiance.

Primary photochemistry, i.e., the photochemical process until the first acceptor of electron, Quinone A (Q_A_), was partly analyzed through the JIP parameters V_J_, N and S_M_. An increase in V_J_ of 46, 53 and 85% was obtained after subjecting the cyanobacterium to control PAR-red light (120 µmol of photon·m^−2^·s^−1^), increased white light (250 µmol of photon·m^−2^·s^−1^) or PAR-red light irradiances (450 µmol of photon·m^−2^·s^−1^), respectively, indicating an increase in the fraction of Q_A_ in its reduced state (Q_A_^−^/Q_A_). Similarly, after subjecting *A. variabilis* cells to PAR-red light or increased PAR-red light, an increase of 77 and 450%, respectively, was obtained in the so-called turn-over number N, which represents the number of times Q_A_ becomes reduced till maximum fluorescence (F_M_) is reached [28]. The increase in V_J_ together with N reflects the accumulation of Q_A_^−^ [29], therefore indicating a possible overreduction of Q_A_ as a consequence of the increase in light irradiance. Similarly, the increase in S_M_ of 60 and 350% in cultures grown on PAR-red light and increased PAR-red light irradiance, respectively, indicates a possible increase in the energy needed to close all reaction centers [28]. Thus, the results obtained seem to reinforce the evidence of an overreduction of Q_A_ when *A. variabilis* is grown on increased PAR-red light irradiances. Nevertheless, interestingly, N and S_M_ values were not affected after increasing white light irradiance, evidencing similar photochemical pressure on the electron transport chain as in control culture.

F_V_/F_0_ is a chlorophyll fluorescence-derived parameter reflecting the proper functioning of PSII [28], a component of the photosynthetic electron transport chain that is particularly sensitive to various abiotic stresses [30]. F_V_/F_0_ expresses the ratio between the rate constants of photochemical and non-photochemical deactivation of excited Chl molecules. F_V_/F_0_ decreases by 19 and 75% after subjecting *A. variabilis* to PAR-red light and increasing PAR-red irradiances, respectively, while a slight increase of 19% was obtained under increasing white light irradiances. Nevertheless, the energy dissipated by the reaction center (DI_0_/RC) was only affected under increasing PAR-red light irradiance. In this sense, the decrease in F_V_/F_0_ together with the increase in DI_0_/RC under increasing PAR-red light suggests, as hypothesized above, enhanced non-photochemical dissipation at the reaction-center level, consistent with photoprotective responses to increased irradiance. The non-photochemical dissipation would be likely mediated by state transitions rather than Orange Carotenoid Protein (OCP)-mediated NPQ, which requires blue-green light for photoactivation. Furthermore, as a defense mechanism against oxidative stress caused by the increase in light irradiance, variation in the content of antioxidant molecules might be expected. Thus, the next section analyzes the variation in antioxidant compounds (carotenoids, phycobiliproteins, polyphenols and flavonoids) and other target bioactive molecules (chlorophylls and lipids) with biotechnological interest, in cultures of the cyanobacterium as a function of light irradiance and quality.

### 2.2. Effect of Light Irradiance and Quality on Accumulation of Target Bioactive Molecules in A. variabilis

This section focuses on partly analyzing the accumulation of target bioactive compounds of the cyanobacterium *A. variabilis* after being subjected to light of different irradiances and qualities. As explained above, the cyanobacterium was grown under repeated-batch mode, in which light irradiance was increased after each cycle, while a culture was maintained with unchanged light irradiance (control culture) for each light quality. Antioxidant molecules such as carotenoids, phycobiliproteins, polyphenols and flavonoids, in addition to other relevant molecules referred to above (chlorophyll and lipids), were determined at the beginning and at the end of the experiment. More details can be found in Section 4.

**Figure 5:**
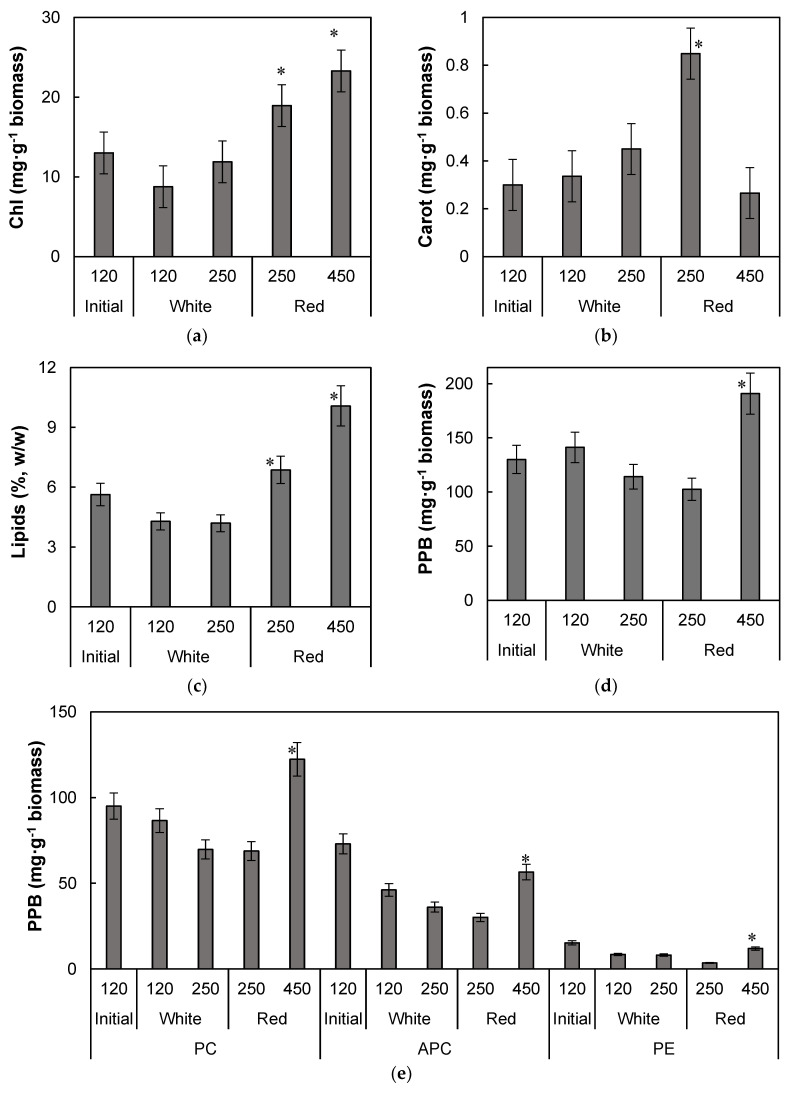
Variation in the intracellular content of (**a**) chlorophylls (Chl), (**b**) carotenoids (Carot), (**c**) total lipids and (**d**,**e**) phycobiliproteins (PPB)—phycocyanin (PC), allophycocyanin (APC) and phycoerythrin (PE)—in *A. variabilis* cultures as a function of light irradiance and quality. X-axis legend: light quality (initial—day 0—or white or red irradiances at day 5; top legend); light irradiance (expressed in µmol of photon·m^−2^·s^−1^; bottom legend), and phycobiliprotein ((**e**), phycobiliprotein content, bottom legend). (*) Represents the significant differences of all treatments with respect to initial data with 95% confidence. More details can be found in Section 4.

Figure 5 shows the variation in the intracellular content of total lipids and photosynthetic pigments (chlorophylls, carotenoids and phycobiliproteins) in *A. variabilis* cells as a function of light irradiance and quality reaching the culture surface. By analyzing the figure, a similar pattern was obtained for both lipids and chlorophyll, with an increase in their content after exposing *A. variabilis* to PAR-red light (Figure 5a,c). The maximum contents, about 10% *w*/*w* and 23.3 mg·g^−1^ biomass, respectively, were obtained in the culture exposed to increased PAR-red light irradiances (450 µmol of photon·m^−2^·s^−1^), representing an approximately 2-fold increase compared to cultures grown under the same irradiance of white light (120 µmol of photon·m^−2^·s^−1^).

In contrast, regarding carotenoids (Figure 5b), no significant variations were observed after varying light irradiance and quality, except for the culture exposed to control PAR-red light irradiance, which showed a content of 0.85 mg·g^−1^ biomass, 4-fold higher than that of white light.

In the case of phycobiliproteins (Figure 5d), similar values as the initial conditions were obtained irrespective of light quality and irradiance, with the exception of the culture exposed to increased PAR-red light irradiance, which reached values of 190 mg·g^−1^ biomass, 1.8-fold higher than those obtained under white light. This increase was mainly due to phycocyanin (123 mg·g^−1^ biomass) and, to a lesser extent allophycocyanin (56 mg·g^−1^ biomass) (Figure 5e).

**Figure 6:**
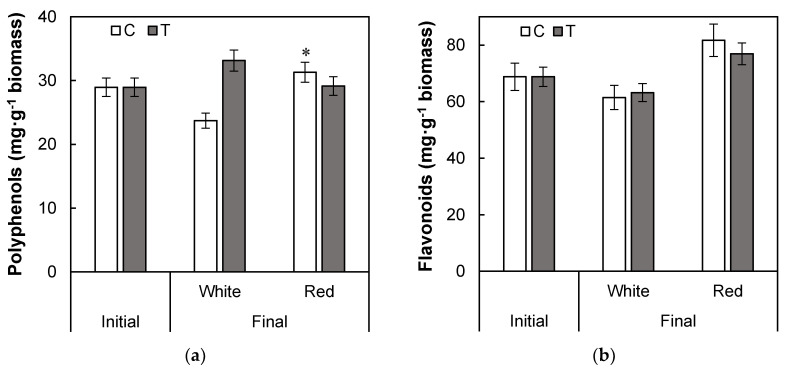
Variation in the intracellular content of (**a**) polyphenols and (**b**) flavonoids in *A. variabilis* cultures as a function of light irradiance and quality. X-axis legend: light quality (white or red; top legend) and cultivation time (initial –day 0– or final –day 5–; bottom legend). (*) Represents the significant differences of all treatments with respect to initial data with 95% confidence.

Figure 6 analyzes the variation in the intracellular content of polyphenols and flavonoids in *A. variabilis* as a function of light irradiance and quality. No significant changes were observed in the content of the above-mentioned phenolic molecules. A slight increase was obtained in polyphenols in the culture exposed to increased white light irradiances, whereas in the case of flavonoids the highest value was obtained under PAR-red light irrespective of the light irradiance.

Light irradiance and quality induce changes in the oxidative status of *A. variabilis*. Among other biochemical responses, the direct consequence of that fact would be the activation of the antioxidant system of the cyanobacterium, through modulation of the content of the target bioactive molecules observed in this section, to counteract the possible production of ROS in the cells. These findings will be further discussed and related to photosynthetic performance in Section 3.

## 3. Discussion

This paper analyzes the growth, photosynthetic performance and non-enzymatic antioxidant system of *A. variabilis* subjected to increased light irradiances and different light quality. As explained above, light irradiances were selected to ensure the viability of the cultures during the experiment. In this sense, the cyanobacterium demonstrated a greater capacity to cope with higher PAR-red light irradiances than white irradiances. For this reason, the selected light irradiances were not in the same range for both light qualities. Indeed, it should be noted that the fact that white and PAR-red light were not evaluated at identical irradiance ranges limits direct spectral-quality comparisons. The apparent advantages of PAR-red light should be interpreted within the context of the irradiance ranges tested for each condition, rather than as a spectral superiority independent of intensity. The paired comparison at 250 µmol photon·m^−2^·s^−1^ (Figure S1) provides a partial spectral-specific reference, comparing JIP parameters related to energy fluxes of the different treatments tested, normalized by its relative control culture, under both light qualities. The absence of significant differences between the PAR-red control (250 µmol of photon·m^−2^·s^−1^) and white light treatment at that same irradiance supports a spectral-quality independency of the response.

### 3.1. Effect of Light Irradiance and Quality on Growth and Photosynthetic Viability of A. variabilis

The results obtained suggest that PAR-red light seems to be more advantageous for *A. variabilis* as the maximum biomass productivities data were obtained earlier than in the cultures exposed to white light, i.e., cultures grown on PAR-red light obtained the higher biomass productivities during the second cycle of repeated-batch cultivation, while under white light the values were obtained during the third cycle. The higher biomass productivities obtained after exposing *A. variabilis* to PAR-red light could be explained based on the literature presented in Section 1, as photosynthetic activity is dependent on the radiation wavelength to which cultures are exposed [31]. For instance, red light has a narrow spectrum between 600 and 700 nm, which corresponds to the absorption wavelength of chlorophyll (665–680 nm), phycocyanin (610 and 665 nm) and allophycocyanin (650 nm) [32]. In this sense, due to the proximity of the red-light spectrum to light absorption complexes in cyanobacteria, red light can be absorbed more efficiently, minimizing energy losses and, therefore, leading to greater productivities [33]. This is similar to the results obtained by Polyzois et al. [34], who studied the effect of light quality in the cyanobacterium *Nostoc* sp. ATCC 53789 and reported that an orange-red filter contributed to a noticeable increase in growth. Similarly, a greater biomass productivity was reported in an extremophilic cyanobacterium *Chroococcidiopsis* sp. when cultivated under PAR-red light compared to white light [35].

Regarding light irradiance, higher biomass productivity was obtained in control culture cells (the lowest light irradiance tested). This fact was also considered beneficial for the optimal production of the cyanobacterium, as high light irradiances are not required for growth. Light availability is one of the major challenges in the large-scale production of photosynthetic organisms, i.e., with increasing cell density, light attenuates rapidly, cells begin to self-shade, and less light is available per cell [36]. This is the reason why having a lower light irradiance requirement results in a more advantageous process for large-scale production, contributing to making the process less energy-consuming and, therefore, more economically viable.

Regarding photosynthetic efficiency of PSII (F_V_/F_M_), the quantum yield (Qy) values obtained in Figure 2 were within the range of 0.2–0.4, indicative of maintenance of functional PSII photochemical activity in cyanobacteria [37], suggesting that the photosynthetic machinery of *A. variabilis* possesses mechanisms to cope with increasing light irradiances and changes in light quality. Nevertheless, some of the Qy values obtained during the first cycle of repeated-batch cultivation (C1) were below that range. Considering that the cyanobacterium has usually been cultivated under white light irradiances of about 120 μmol photon·m^−2^·s^−1^, the above-mentioned decrease in Qy values could be partly attributed to the adaptation of the cyanobacterium to the shifted spectral quality. Thus, the transient decrease in F_V_/F_M_ during C1 under PAR-red light is attributed to the physiological cost of adapting from prior white light conditions to a new spectral quality.

Accordingly, by analyzing the selected JIP parameters studied in Figure 3 and Figure 4, it could be deduced that the photosynthetic machinery of *A. variabilis* was more affected after increased PAR-red light irradiances. As explained above, increased PAR-red light was expected to eventually cause greater oxidative stress on *A. variabilis* cells. Despite that fact, F_V_/F_M_ values were not affected under these conditions and, interestingly, biomass productivity increased. In this sense, it becomes evident that the cyanobacterium must express mechanisms to efficiently cope with increasing light irradiances, and the change in light quality should have a role in it.

By analyzing the results obtained in Figure 3, it can be deduced that different light irradiances and qualities impact the photosynthetic machinery of *A. variabilis*. That is, a significant increase in ABS/RC and DI_0_/RC was obtained as light irradiance increases, irrespective of the light quality (white or red), while, interestingly, slights variations were obtained in TR_0_/RC and ET_0_/RC with respect to the control culture. These variations were greater after exposing *A. variabilis* to increasing PAR-red light irradiances. Nevertheless, the activation of photoacclimation mechanisms, or a related response, is evidenced by both white and red light; i.e., despite the increase in light irradiance (ABS/RC), the cyanobacterium was able to maintain the energy flux transduced in the electron transport chain (ET_0_/RC) unchanged by partially dissipating excess energy (DI_0_/RC). By doing so, oxidative pressure may be limited and, consequently, ROS formation may also be reduced [38].

In turn, after exposing *A. variabilis* to increasing light irradiances, especially PAR-red irradiance, the considerable increase in V_J_ and N suggests an overreduction of the primary quinone acceptor, indicating a possible increase in the oxidative pressure on the ETC [39]. Interestingly, N and S_M_ values were not affected after increasing white light irradiances, unlike increasing PAR-red light irradiance, which had a greater effect on the photosynthetic machinery of the cyanobacterium, under the conditions tested. Indeed, under increased PAR-red light irradiance, the photosynthetic machinery appears more affected, as reflected by higher ABS/RC and DI_0_/RC values. However, the energy dissipation observed (DI_0_/RC increase) is unlikely to involve OCP-mediated non-photochemical quenching (NPQ), since this mechanism requires blue-green light (400–550 nm) for OCP photoactivation [40], and would not be induced under the 600–700 nm conditions used in our study. A more consistent explanation is that (i) PAR-red light coincides with the absorption maxima of phycocyanin (620 nm) and allophycocyanin (650 nm), enhancing energy-transfer efficiency through the PPB cascade and reducing excitation-energy leakage at intermediate steps; and (ii) state transitions (redistribution of PPB between PSI and PSII driven by PQ pool redox status) provide the regulatory mechanism for non-photochemical energy dissipation under red light conditions. Together, these processes may limit the net excitation pressure on the electron transport chain and thereby potentially reduce ROS formation.

Under white light, irrespective of the light irradiance, energy flux data showed no significant differences (Figure S1). In this sense, under a 2-fold increase in white light irradiance, the cyanobacterium seemed to apparently reduce photooxidative damage by expectedly absorbing only the energy that can be processed by the ETC, indeed resulting in similar ABS/RC and ET_0_/RC values irrespective of the white light irradiance [38]. This conclusion is similar to that obtained by Srilatha et al. [41], who analyzed the 48 h effect of high light on *Chlamydomonas reinhardtii* cultures and obtained faster growth, likely promoted by acclimation to high irradiance levels through increased NPQ for excess light dissipation as a photoprotective mechanism.

As discussed above, after exposing *A. variabilis* to increasing PAR-red light irradiance, the photosynthetic machinery appears to be more affected, as higher N and S_M_ values were obtained, indicating greater overreduction of Q_A_, which might eventually lead to ETC saturation and photoinhibition [29]. Furthermore, energy dissipation by the reaction center (DI_0_/RC) was only increased under increasing PAR-red light irradiance. In this sense, the increase in DI_0_/RC under increasing PAR-red light suggests, as hypothesized above, energy-transfer efficiency through the PPB cascade, and enhanced non-photochemical dissipation via state transitions, consistent with photoprotective responses that may limit excess energy pressure on the ETC [28]. Accordingly, the improved biomass productivities for *A. variabilis* cultures grown on PAR-red light, irrespective of irradiance and under the conditions tested, point to the effective functioning of photoacclimation mechanisms in *A. variabilis* to cope with increases in light irradiances.

Furthermore, as a defense mechanism against oxidative stress caused by increased light irradiance, variation in the content of antioxidant molecules would be expected to occur. Thus, the next section analyzes the variation in antioxidant compounds (carotenoids, phycobiliproteins, polyphenols and flavonoids), in addition to chlorophylls and lipids, with biotechnological interest, as a function of light irradiance and quality.

### 3.2. Effect of Light Irradiance and Quality on Accumulation of Target Bioactive Molecules in A. variabilis

Increasing light irradiance and variation in light quality have been proven to cause oxidative stress in the cyanobacterium *A. variabilis*. By analyzing the variation in target antioxidant molecules of *A. variabilis*, an eventual role of the antioxidant system to partly cope with the oxidative stress also became evident.

Interestingly, increasing white light irradiance has no effect on the accumulation of antioxidant compounds. Thus, it would seem that photoacclimation performed by the photosynthetic machinery is sufficient to cope with the white light irradiance imposed. However, under the conditions tested, PAR-red light has an impact on the variation of antioxidant compounds in the cyanobacterium, especially increasing PAR-red light irradiance, which was the growth condition that most affected the photosynthetic performance of *A. variabilis*. This could be justified, as explained above, by the fact that increasing PAR-red light was expected to cause greater stress on *A. variabilis* cells.

Increasing PAR-red light irradiances—those tested in this study—led to an enhancement in the biosynthesis of total lipid content (Figure 5). Light is one of the vital abiotic factors influencing the composition of biomass constituents in microalgae [18]. Thus, increased irradiances generate an increase in pressure in the ETC, as partly evidenced in the previous section. The primary direct consequence can be an increase in reducing equivalents, NAD(P)H [4]. Part of this chemical energy might be oxidized by ROS formed in the chloroplast [42]. One of the metabolic strategies of microalgae to dissipate excess reducing power, i.e., chemical energy derived from the photosynthesis, is to divert excess NAD(P)H to the synthesis of triacylglycerides (TAG); using this strategy, microalgae can store carbon and energy in the form of fatty acids [43].

This could partly explain the increased lipid content in *A. variabilis* when cultured under increased PAR-red irradiances. However, it is worth noting the large increase obtained under the conditions referred to above in phycobiliproteins and chlorophylls (Figure 4 and Figure 5), the two main photosynthetic pigment families in cyanobacteria [12]. In this scenario, a readjustment of the photosynthetic machinery to cope with the excess electron flux could be required, which partly suggests that the increase in lipids may not only counteract ROS generation, but also may contribute to enhancing the number of antenna complexes required for coping with increased photon flux. As described, cyanobacteria have developed several light acclimation strategies to adapt to changes in light quality and intensity, termed chromatic acclimation (CA), which involves wavelength-dependent adjustment in the protein and/or pigment composition of PPB [27]. For instance, several authors have reported an increase in phycocyanin (PC) and allophycocyanin (APC) under PAR-red light irradiance compared to other light qualities [44,45]. This can be justified considering that the absorption peaks of PC (~620 nm) and APC (~650 nm) [32] fall within the emission spectrum of red LEDs (600–700 nm), thus favoring light absorption for photosynthesis. Additionally, visible light energy that is poorly utilized by chlorophyll *a* can be efficiently absorbed by PBP and transferred to the photosynthetic reaction centers [46], as explained.

Indeed, phycobilisomes not only facilitate efficient light capture but also contribute to photoprotection via mechanisms such as non-photochemical dissipation [46]. Delgado et al. [47] described that increased total PBP accumulation reflects a physiological adjustment aimed at maximizing light utilization while mitigating photodamage. These findings are consistent with the JIP parameter variations obtained in this study (Figure 3 and Figure 4), where *A. variabilis* maintained the electron transport flux (ET_0_/RC) unchanged despite increased PAR-red light irradiance. Analysis of chlorophyll fluorescence (JIP parameters) revealed that the photosynthetic machinery adapts to increased irradiance by modulating energy fluxes. PAR-red light promotes dissipation of excess absorbed energy at the phycobilisome level, independently of and complementarily to, the increase in light-harvesting antenna pigments, thereby reducing the net oxidative pressure within the electron transport chain.

The ratio PC:PE:APC (normalized to APC) is species-dependent in cyanobacteria [32]. *S. platensis* was reported to produce 127.5 mg·g^−1^ of total PBPs under approximately 27 μmol photon·m^−2^·s^−1^ [48], in a ratio of 2.25:0.5:1. The ratio of *Chroococidiopsis* sp. under different light irradiances varies between 1.14:0.65:1 (APC = 73.0 mg g^−1^) under 10 μmol photons m^−2^ s^−1^ to 0.32:0.28:1 (APC = 95 mg g^−1^) under 150 μmol photons m^−2^ s^−1^ [32]. Similarly, under white light of 25 μmol photon m^−2^·s^−1^, *Leptolyngbya* sp. showed a ratio of 1.05:1.78:1 (APC = 28 mg g^−1^), whereas at 100 μmol photon m^−2^·s^−1^ the ratio was 0.91:0.85:1 (APC= 23 mg· g^−1^) [49]. Both species obtained their highest PPB content under low light irradiance, PE being the most abundant. Conversely, in this study, the ratio in *A. variabilis* was 2.16:0.21:1 under the increased PAR-red light irradiances tested and 1.88:0.18:1 under standard white light conditions (Table 1), indicating a shift toward PC enrichment under red light. These results suggest that *A. variabilis* adjusts its pigment composition in response to light conditions, with the predominance of PC, which is consistent with its central role in cyanobacterial light-harvesting systems [32].

Several bioactivities, including antioxidant, anticancer, neuroprotective, anti-inflammatory, hepatoprotective and hypocholesterolemic effects, have been reported for phycobiliproteins and other accessory pigments [21,22]. It is worth noting the content of PPB obtained in *A. variabilis* under increased PAR-red light irradiance of 123 mg·g^−1^ biomass of PC, 56 mg·g^−1^ biomass of APC, and 12 mg·g^−1^, which enhance the potential of *A. variabilis* biomass for its biotechnological potential. Phycocyanin has been reported to scavenge free radicals with potent antioxidant, anti-inflammatory and anticancer properties, and to inhibit cell proliferation of human leukemia K562 cells [32]. Allophycocyanin (APC) inhibits enterovirus 71-induced cytopathic effects [50]. Phycoerythrin (PE) has been described to display antitumor activity against human liver carcinoma cells SMC 7721, and recently its anti-Alzheimeric potential has been reported [51]. In addition to medical applications, PBP is used in food and cosmetic industry [32], although for health applications PBP from *A. variabilis* (or cultures of the cyanobacterium) should be proven to be free of toxins. Indeed, as is mandatory, before planning any potential commercial use of microalgae or cyanobacteria biomass for food, nutraceutical, cosmetic or pharmaceutical applications, rigorous strain-level safety validation is required. This must include, at least, among other tests, genomic screening for the gene clusters associated with cyanotoxin production, targeted toxin quantification by LC-MS/MS, and in vitro cytotoxicity assays on the final biomass preparation.

Regarding carotenoids, since their absorption spectrum does not overlap with the red-light emission range (~450 nm) [17], the apparent reduction obtained under increased PAR-red light could be partly attributed to their role as ROS scavengers rather than their photosynthetic performance [18]. Carotenoids are antioxidant molecules, and microalgae increase their intracellular concentration to eliminate excess free radicals derived from oxidative stress [52]. Nevertheless, several authors have described structural changes in antioxidant molecules after reacting with ROS, which would affect their detection procedure. For instance, ROS, especially singlet oxygen, produced in chloroplasts under increased oxidative pressure, can oxidize β–carotene, leading to the production of various oxidized derivatives, such as β–cyclocitral or β–ionone, containing α,β-unsaturated carbonyl groups [53,54]. Interestingly, non-significant variations were obtained in phenolic compounds, suggesting their constitutive presence in *A. variabilis*.

The lack of significant variation in polyphenols and flavonoids may also suggest that photoacclimation and state-transition-mediated energy dissipation were sufficient to prevent the sustained oxidative stress required to induce these secondary antioxidant pathways at the irradiances tested.

Overall, this study represents an attempt to analyze how *A. variabilis* responds to variations in light irradiance combined with different light spectral ranges, particularly regarding antioxidant and photoacclimation responses. *A. variabilis* not only tolerates increased light irradiances and different light qualities but also enhances biomass productivity under increased PAR-red light. The photosynthetic machinery of the cyanobacterium has been demonstrated to adapt to increasing light irradiances by modulating the energy flux that is transduced to photosynthesis in the ETC, by dissipating part of the excess energy absorbed, which is consistent with a photoprotective adjustment that may limit excess excitation pressure and thereby potentially reduce ROS formation.

It is important to distinguish between two complementary aspects of photosynthetic performance: light-harvesting capacity, reflected in increased pigment content (chlorophylls and phycobiliproteins, Figure 5a,d), and photochemical efficiency per reaction center, reflected in F_V_/F_M_ (Figure 2b). Under increased PAR-red light, *A. variabilis* primarily enhanced its absolute light-harvesting capacity through PPB expansion, while the higher photon flux simultaneously increased the fraction of closed PSII reaction centers, explaining unchanged or slightly reduced F_V_/F_M_ relative to white light controls. This pattern indicates that PAR-red light—within the irradiance range tested—acts mainly as a stimulus for antenna expansion and biosynthetic productivity, rather than a direct enhancer of photochemical efficiency, a distinction that is important for the integrated interpretation of Figure 4 and Figure 5. Additionally, the antioxidant system contributes to stress mitigation, as evidenced by increased lipid and, partly, pigment content. This may reflect both photoprotective responses and enhanced light-harvesting capacity. These results highlight *A. variabilis* as a promising candidate for production of high-value antioxidant compounds, particularly phycobiliproteins such as phycocyanin, whose content increased 30% under increased irradiances and is known for its potent bioactive properties.

## 4. Materials and Methods

### 4.1. Biological Material and Culture Conditions

The cyanobacterium *Anabaena variabilis* ATCC 29413 (*A. variabilis*) was used as the model organism in this study. The strain was obtained from the culture collection of the Biotechnology of Extremophiles Laboratory, University of Huelva (Spain). Cultures were grown in a culture medium based on NPK-based fertilizer media; the media composition was as follows, per liter: 0.0155 g NO_3_^−^, 0.00812 g NH_4_^+^, 0.0568 g P_2_O_5_ y 0.0603 g K_2_O and 0.4 mL of micronutrient solution (Microfer Complex, Fercampo S. A., Málaga, Spain). Cultivation was performed in flat glass bottles with a final working volume of 1 L. Culture conditions were maintained at 25 ± 2 °C under continuous sparging with air enriched with 2.5% (*v*/*v*) CO_2_ to maintain homogeneous cell suspension. Illumination was provided using LED systems under white light (WL) and red light within the PAR range (600–700 nm), with irradiance expressed as μmol photon·m^−2^·s^−1^. Illumination was provided using LED systems (Philips, Amsterdam, The Netherlands): white light by broad-spectrum white LED panels (380–780 nm, peak 450 nm), and red light by narrow-band red LED panels (peak 660 nm, PAR range 600–700 nm). Irradiance was measured at the outer surface of the glass bottles using a calibrated quantum sensor, and values are expressed as µmol photon·m^−2^·s^−1^. LED panels were positioned 15 cm from the bottle surface. The culture setup is shown in Figure S2.

Cultivation was performed under a *repeated-batch* regime with three consecutive cycles (C1–C3), maintaining optical density within the range of OD_750_ = 1.5–3.0 through periodic dilution to prevent self-shading. For each light condition, control cultures (constant irradiance) and treatment cultures with stepwise increases in irradiance between cycles were established, as detailed in Table 2.

### 4.2. Growth and Biomass Productivity Determination

Growth was followed by measuring optical density (OD) of the cultures at 750 nm in a Cary 60 UV–Vis spectrophotometer (Agilent Technologies, Santa Clara, CA, USA) equipped with a temperature control system adjusted to 25 °C. The OD_750_ data expressed in absorption units (AU) provided information on culture turbidity, which is proportional to the amount of biomass present in the culture [55].

Productivity, the increase in biomass in a culture over time, was calculated through the following equation:

(1) $$Productivity\left(g\cdot L^{-1}\cdot d^{-1}\right)=\frac{C_{t}-C_{0}}{t_{t}-t_{0}}$$

where $C_{t}$ and $C_{0}$ represent the cell density for times *t* and zero. Cell density was determined by measuring the dry weight (dw) of the biomass contained in 2 mL of culture medium.

### 4.3. Photosynthetic Performance Determination

Photosynthetic performance of the cyanobacterium species after exposure to different light irradiance and quality were measured with the portable PAM fluorimeter AquaPen-C AP-C 100 (Photon Systems Instruments, Brno, Czech Republic) using the FluorPen 1.1.2.6 software to access the data. Culture samples were diluted so that all Chl fluorescence measurements were performed on samples with the same cell concentration, OD_750_ = 0.2 (OD_750_, optical density at 750 nm). The OJIP test was performed in cells adapted to the dark for 15 min. The typical polyphasic OJIP curve provides information on the kinetics and heterogeneity involved in the reduction of plastoquinone (PQ) and Q_A_, which is the primary electron acceptor [28]. In addition, by analyzing the Chl fluorescence transients of the OJIP test, different JIP parameters could be calculated and discussed throughout the text. The JIP test [28] provides the following parameters: maximum quantum yield of PSII (F_V_/F_M_); absorption flux per active RC (ABS/RC); trapped energy flux per RC (TR_0_/RC); electron transport flux per RC (ET_0_/RC); dissipated energy per RC (DI_0_/RC); relative variable fluorescence at the J step (V_J_); turn-over number Q_A_ (N); normalized area (S_M_); and maximum quantum yield of photochemistry of PSII (F_V_/F_0_).

### 4.4. Chlorophyll and Carotenoid Determination

Chlorophyll (Chl) content was determined as described by Robles et al. [5]. Culture samples containing 1 or 2 mL were centrifuged at 3.0 RCF for 5 min in a Minispin model (Eppendorf, Hamburg, Germany); methanol was added to the pellet and the mixture was placed in an ultrasound Ultrasons H-D 3000866 (Selecta, Barcelona, Spain) model bath with a power of 330 W at 60 °C for 5 min to weaken the microalgal cell wall. After another centrifugation step, the supernatant was collected and analyzed by UV/Visible spectrophotometry. Modified Arnon’s equations were used to calculate chlorophyll and carotenoid concentrations in the extracts.

### 4.5. Phycobiliprotein Determination

Phycobiliprotein content was extracted with 1 mL of phosphate buffer (0.1 M; pH 7) and subjected to an ultrasonic bath for 2 h at 30 °C. Then, the samples were centrifuged at 14,000× *g* for 10 min, and the supernatant was transferred to a clean Eppendorf tube for measurement of phycobiliprotein content. The absorbance was measured at 565 nm, 620 nm, and 650 nm. Subsequently, the phycobiliprotein concentration in the biomass pellets was calculated using Bryant’s equations and expressed in mg mL^−1^ of extract, as described by Bryant et al. [56]. In this analysis, PC corresponds to phycocyanin, APC to allophycocyanin, PE to phycoerythrin, and A represents the absorbance recorded at the respective wavelengths.

(2) $$PC=\frac{A_{620}-0.72\;A_{650}}{6.29}$$

(3) $$APC=\frac{A_{650}-0.191\;A_{620}}{5.79}$$

(4) $$PE=\frac{A_{565}-2.41\;CPC-1.41CAPC}{13.02}$$

### 4.6. Lipid Determination

Total lipids were extracted following the procedure described by Axelsson et al. [57]. To do this, 50 mg of dry biomass was weighed into glass vials and 8 mL of a chloroform/methanol solution (2:1, *v*/*v*) was added. The mixture was vortexed to promote lipid extraction and then 2 mL of a 0.73% (*w*/*v*) NaCl solution was added to induce phase separation. The samples were centrifuged at 3.0 RCF for 5 min and the lower phase (chloroform) was carefully recovered in previously tared vials and evaporated at 100 °C for 24 h. The remaining residue was cooled in a desiccator before being weighed again. The total lipid content (L) was determined gravimetrically using Equation (5), where $P_{f}$ is the final weight, $P_{i}$ is the initial weight and biomass is the amount of dry biomass.

(5) $$L\left(\%\;w/w\right)=\frac{P_{f}\left(g\right)-P_{i}(g)}{biomass(g)}\times 100$$

### 4.7. (Poly)phenolic Compound Determination

Total (poly)phenols were determined using the procedure described by Georgé et al. [58]. Accordingly, (poly)phenolic compounds were oxidized by the Folin–Ciocalteu reagent (Panreac, Barcelona, Spain). The reaction was carried out in an alkaline medium; for this, sodium carbonate was added to the methanolic samples obtained in Section 4.4, and the absorbance was measured at 725 nm by UV–Vis spectrophotometry, model Evolution 201 (Thermo Fisher Scientific, Walthman, MA, USA). Total (poly)phenolic content was determined as mg-eq gallic acid·L^−1^ using the equation obtained from a standard gallic acid 1-hydrate (Panreac, Barcelona, Spain) calibration curve.

Flavonoid compounds were determined by a spectrophotometric method described by Liu et al. [59]. The absorbance value was measured at 510 nm using UV–Vis spectrophotometry, model Evolution 201 (Thermo Fisher Scientific, Walthman, MA, USA). Total content of flavonoids was calculated as mg-eq catechin·L^−1^ using the equation obtained from a standard catechin, (+)-catechin hydrate (Sigma Aldrich, Darmstadt, Germany), calibration curve.

### 4.8. Statistics

Unless otherwise stated, data are presented as the mean of three independent replicates, and standard deviations are shown in the corresponding figures and tables. Data from the different treatments were analyzed using univariate statistical models and analysis of variance (ANOVA), considering a 95% confidence level for the determination of statistical significance. All statistical analyses were performed using the software Jamovi (version 2.3; The Jamovi project, 2024).

## Figures and Tables

**Table 1:** Ratio of PC:PE:APC (normalized to APC) in *A. variabilis* cultures as a function of light quality and irradiance.

Light Quality	Light Irradiance (μmol photon·m^−2^·s^−1^)	PC:PE:APC
White	120	1.88:0.18:1
	250	1.93:0.23:1
Red	250	2.29:0.12:1
	450	2.16:0.21:1

**Table 2:** Experimental irradiance conditions for *A. variabilis* grown under white and PAR-red light in repeated-batch mode. Cultures were subjected to three consecutive cycles (C1–C3). Control cultures (C) were maintained at constant irradiance, whereas treatment cultures (T) experienced stepwise increases in irradiance between cycles. Irradiance values are expressed as μmol photon·m^−2^·s^−1^.

Repeated-Batch Cycle	White Light		Red Light	
	Control (C)	Treatment (T)	Control (C)	Treatment (T)
C1	120	120	250	250
C2	120	185	250	350
C3	120	250	250	450

## Data Availability

The original contributions presented in this study are included in the article/Supplementary Materials. Further inquiries can be directed to the corresponding author.

## Supplementary Materials

The following supporting information can be downloaded at: https://www.mdpi.com/article/10.3390/md24060221/s1, Figure S1: JIP parameter variation normalized by the control culture for each light irradiance tested on *A. variabilis* cultures during the last cycle of repeated-batch cultivation; Figure S2: Experimental setup of *A. variabilis* cultures.
