# Supplementary material for: Red Light Enhances Biomass and Bioactive Compounds Through Photosynthetic Acclimation in Anabaena variabilis
Source: Mar Drugs. 2026 Jun 19;24(6):221. doi: 10.3390/md24060221 (PMC13302619; doi:10.3390/md24060221)
Supplement: Supplementary file 1 [file marinedrugs-24-00221-s001.zip › marinedrugs-4334334-supplementary.pdf]

Article

Red Light Enhances Biomass and Bioactive Compounds through Photosynthetic Acclimation in *Anabaena variabilis*

Carol Ostojic [1,2], María Robles [1,*], Lidia Martín-Gordillo [1], David Fernández [1], Riccardo Gava [2] and Carlos Vílchez [1]

[1] Biotechnology of Extremophiles Lab, CIQSO-Centro de Investigación en Química Sostenible and CIDERTA, Faculty of Experimental Sciences, University of Huelva, 21071, Huelva, Spain; maria.robles@dqcm.uhu.es (M.R.); lidia.martin@dqcm.uhu.es (L.M.); frdavid14@gmail.com (D. F.)

[2] Bioplagen S.L., Av. Castilleja de la Cuesta, 20-22, Bollullos de la Mitación, 41110 Seville, Spain; riccardogava@bioplagen.com

* Correspondence: maria.robles@dqcm.uhu.es (M.R.); cvilchez@uhu.es (C.V.)

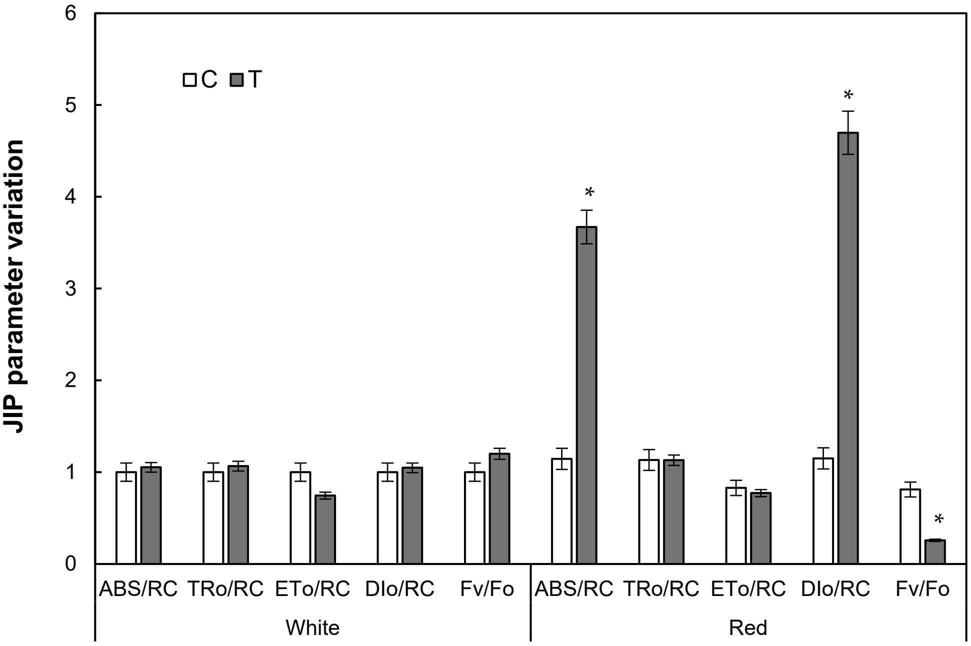

Figure S1. JIP parameter variation normalized by the control culture (without light irradiance increase) for each light irradiance tested on *A. variabilis* cultures during the last cycle of repeated batch cultivation (cycle 3, C3). (*) Represents the significant differences of all treatments with respect to control culture data with 95% confidence.

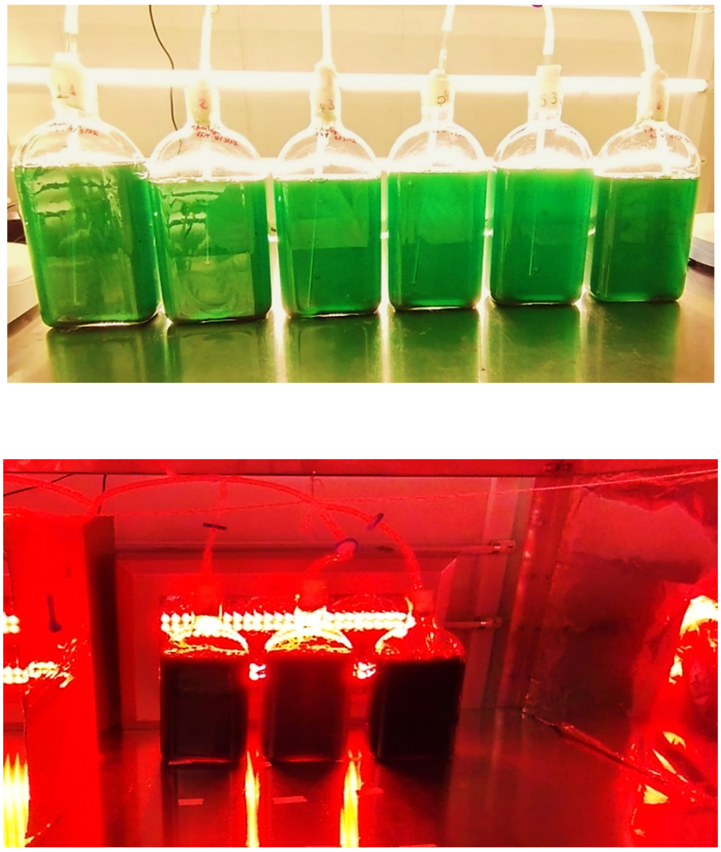

Figure S2. Experimental setup of *A. variabilis* cultures. Top: cultures growing under white-light LED illumination; Bottom: cultures growing under red-light LED illumination.
